# Hot water immersion; potential to improve intermittent running performance and perception of in-game running ability in semi-professional Australian Rules Footballers?

**DOI:** 10.1371/journal.pone.0263752

**Published:** 2022-02-16

**Authors:** Calvin P. Philp, Nathan W. Pitchford, James W. Fell, Cecilia M. Kitic, Martin Buchheit, Aaron C. Petersen, Christopher T. Minson, Denis C. Visentin, Greig Watson

**Affiliations:** 1 Sport Performance Optimisation Research Team, School of Health Sciences, University of Tasmania, Launceston, Tasmania, Australia; 2 Western Bulldogs Football Club, Footscray, Victoria; 3 School of Health, Medical and Applied Sciences, Central Queensland University, Brisbane, Queensland, Australia; 4 French National Institute of Sport (INSEP), Laboratory of Sport, Expertise and Performance (EA 7370), Paris, France; 5 Institute for Health & Sport, Victoria University, Melbourne, Victoria, Australia; 6 HIITScience, Revelstoke, British Columbia, Canada; 7 Kitman Labs, Performance Research Intelligence Initiative, Dublin, Ireland; 8 Department of Human Physiology, University of Oregon, Eugene, Oregon, United States of America; Universiti Malaya, MALAYSIA

## Abstract

This study investigated whether hot water immersion (HWI) could heat acclimate athletes and improve intermittent running performance and perception of in-game running ability, during a competitive Australian Rules Football (ARF) season. Fifteen male semi-professional ARF athletes (Mean (SD); age: 22 (3) years, height: 182.3 (6.5) cm, mass: 80.5 (5.1) kg) completed either HWI (HEAT, N = 8, 13 (2) sessions, 322 (69) min exposure, 39.5 (0.3) °C) or acted as a control (CON, N = 7, no water immersion) over 6-weeks. Athletes completed a 30–15 Intermittent Fitness Test pre and post-intervention to assess intermittent running performance (V_IFT_), with perception of in-game running ability measured. Heat acclimation was determined via change in resting plasma volume, as well as physiological and perceptual responses during HWI. HEAT elicited large PV expansion (mean ± 90% CI: d = 1.03 ± 0.73), large decreases in heart rate (d = -0.89 ± 0.70), thermal sensation (d = -2.30 ± 1.15) and tympanic temperature (d = -1.18 ± 0.77). Large improvements in V_IFT_ were seen in HEAT (d = 1.67 ± 0.93), with HEAT showing a greater improvement in V_IFT_ when compared to CON (d = 0.81 ± 0.88). HEAT also showed greater belief that in-game running ability improved post-intervention (d = 2.15 ± 1.09) compared to CON. A 6-week HWI intervention can elicit heat acclimation, improve perception of in-game running ability, and potentially improve V_IFT_ in semi-professional ARF athletes.

## Introduction

With the increasing competiveness and time demands associated with an Australian Rules Football (ARF) season, practitioners (e.g., performance, sports science and medical staff) are always searching for time-efficient methods to improve running performance, in the hope of enhancing competitive performance. Superior intermittent running performance is associated with higher match possessions and greater high-intensity (>15 km·hr^-1^) match running output in ARF [1]. As such, ways to improve this fitness component are of high interest. Heat acclimation (HA), the process of repeated exposure to high environmental temperatures to elicit favourable physiological adaptations is one such method that has gained increased interest in both ARF and other team sports.

The use of HA in team sport athletes has yeilded promising results, with, to the authors knowledge, four [2–5] out of six [2–7] HA studies showing improvements in running performance ranging from 1.5–44% (d = 0.5–2.0). Of the six studies identified, five [3–7] utilised short-term HA protocols (≤7 sessions), and one utilised a moderate-term (8–14 sessions) HA protocol [2]. The largest improvements were reported in elite ARF players, who increased their intermittent running performance in a temperate environment (23 °C) by 44% (d = 2.0) following a 14-day pre-season HA camp protocol [2]. Despite these promising effects, three out of the six studies identified lacked a control group. Furthermore, all but one were performed either in the pre-season or during the lead up to a major competition, when training volumes are often at their highest and a greater proportion of training is attributed to conditioning and physical development, compared to the in-season [8].

As the Australian Football League competitive in-season period lasts 23 weeks, it is likely that a decrease in aerobic fitness will occur throughout the season given the reduced training load and decreased focus on aerobic fitness development [8]. Therefore, the use of a time-efficent means to improve or maintain intermittent running performance during the in-season period, such as HA, presents as a useful training methodology to enhance performance. For example, Buchheit et al. [3] observed a 7% increase in intermittent running performance in elite footballers following an 11-day HA training camp. However, this study required participants to be flown internationally to a hot, humid environment, something not feasible for many sporting teams, especially in-season. Therefore, a modality that enables favourable physiological adaptations without an increase in training load or travel warrants attention.

Post-exercise hot water immersion (HWI) offers an easily accessible thermal stimulus that has the potential to elicit physiological and performance improvements, whilst providing minimal disturbance to the athletes training program. Unlike active HA protocols that require facilities such as heat chambers or high ambient temperatures, HWI can be implemented through the use of hot baths or spas; facilities accessible to the broader population. Several studies [9–12] have shown physiological adaptation consistent with HA after the use of HWI, however, to date only one study has investigated the effect of HWI on maximal running performance. Zurawlew and colleagues [11] found a 5% (~60-sec) improvement in 5km time-trial performance in trained individuals in hot (33 °C) but not temperate (18 °C) conditions, following six successive days of 40-min post-exercise HWI sessions. Furthermore, as per the protocol utilized by Zurawlew et al. [11], HWI is passive in nature and can be undertaken post training sessions, therefore, miniminsing the risk of decreased training quality and locomotive output that is consistent with exercise [13] and team training [2, 14] in the heat. Although HWI has the potential of being a useful conditioning strategy, to the authors’ knowledge, there is currently no literature investigating the effect of HWI on intermittent running performance in team sport athletes, particularly the effect of intermittent HWI (2–3x/week). Consequently, further research would be of interest to practitioners.

In addition to the physiological benefits, an often overlooked aspect of training studies is an athlete’s belief in the performance benefit of the training intervention. This is despite previous examples of belief-effects with well-acknowledged interventions such as altitude [15], caffeine [16], bicarbonate [17] and cold water immersion [18]. HA is one such modality that may allow practitioners to capitialise on important psychological elements such as increased confidence and the athletes own belief in their preparation. Given that HA has been shown to reduce perception of exercise intensity [19] as well as improve intermittent running performance [2–5], HWI may enable athletes to feel more physically prepared, thus improving belief in physical output and performance. To date, the authors are not aware of any research investigating the athletes perception of HA on their testing and in-game exercise performance.

While there is currently no supporting research for the use of HWI for psychological benefits, and only a small amount supporting running performance benefits (none of which relate to intermittent running performance), HWI may provide a useful strategy for team sport practitioners to implement an effective psychological and conditioning stimulus during a period of the season that has traditionally faced resistance due to increased training load. Therefore, the aim of this study was to investigate whether an intermittent post-exercise HWI of up to 30-min, 2–3x/week following team training can improve in-season intermittent-running performance and perceptions of running ability in semi-professional ARF athletes.

## Materials and methods

### Experimental approach to problem

**Fig 1:**
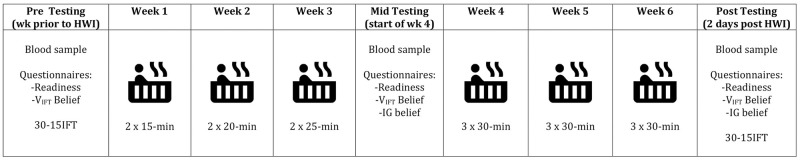
Outline of hot water immersion intervention. 30-15_IFT_: 30–15 Intermittent Fitness Test. V_IFT_ belief: Intermittent running performance belief. IG belief: in-game running ability belief.

This investigation utilised a 6-week parallel-group study design, whereby semi-professional ARF athletes from a single Victorian Football League (VFL) club were allocated to either perform HWI or act as a control. The hot water immersion group (HEAT) performed HWI in a spa for up to 30 min, 2–3x/week following team training, whilst the control group (CON) were instructed to continue with their usual post-training schedules (no water immersion). A graded intermittent running test (30-15_IFT_) [20] was utilised to determine running performance change, as intermittent running performance has previously been associated with match performance in ARF [1]. Belief questionnaires (outlined further below) were collected to identify the psychological impact of the intervention on the participants. Physiological and blood measures consistent with HA were collected to determine if acclimation occurred in the HEAT group. A schematic diagram of the intervention design can be seen in Fig 1.

### Subjects

Seventeen male Victorian Football League ARF players were recruited from the same football club, of which fifteen (Mean (SD) age: 22 (3) yr, height: 182.3 (6.5) cm, mass: 80.5 (5.1) kg completed the intervention. After baseline testing, participants were divided in to HEAT (n = 8, age: 22 (2) yr, height: 183.5 (6.7) cm, mass: 80.8 (5.8) kg or CON (n = 7, age: 22 (3) yr, height: 180.9 (6.6) cm, mass: 80.2 (4.5) kg, with groups matched for intermittent running performance (V_IFT_). Two participants were withdrawn from the study; one due to being promoted to the professional level (HEAT), the other due to injury sustained during competition (CON). Participants provided written informed consent and the study was approved by the Institutional Human Research Ethics Committee (Ethics no. H0013872), which conformed to the recommendations of the Declaration of Helsinki.

### Procedures

#### Intervention

This study was undertaken during the competitive season in Australia, during the months of April–June (average maximum daily environmental temperature during study period was 19 ± 5 °C) [21]. All participants were required to continue normal football training sessions (~three skill sessions, three gym session and one match per week), and to avoid any additional training. Participants in HEAT were scheduled to complete 15 passive immersions, seated submerged above shoulder level in a hot spa (39.5 (0.3 °C)), over six weeks. These HWIs occured approximately 30–45 min after team training or exercise, and the majority occurred between 5:00 p.m. to 8:30 p.m. HWI occured at a similar time of day to the 30-15_IFT_. The intervention consisted of a progressive HWI protocol (Fig 1) to allow HEAT participants to be gradually exposed to the new stimulus. This also minimized the potential of decay in adaptation as a result of a reduced thermoregulatory stimulus suggested with constant load HA protocols [22]. These considerations were deemed important as the study was conducted in-season. HEAT participants followed this protocol unless they were unable to tolerate the hot spa for the allocated duration, or were advised not to by the football club medical team due to injury (e.g., contusions). Throughout the intervention, CON performed their regular post-training routine (included no water immersion) in order to compare to what would regularly occur in the semi-professional environment. This was determined to make the comparison between groups as applicable to the applied setting as possible. For the purpose of reporting, athletes initial HWI immersion session of Week 1 (Initial) and Week 4 (Mid), as well as their final (Final) HWI session were reported as noteable time points. Heart rate, tympanic temperature and thermal sensation [23] were collected for each HWI session. During HWI participants were given access to both water and a commercial sports drink solution. Fluids were consumed ad libitum throughout the HWI, with fluid consumption measured to determine intake. After each HWI session, participants were encouraged to consume 1.5x the fluid lost (based on the assumption that 1g body mass change equalled 1ml fluid loss) during the session. Rating of perceived exertion (RPE) was collected following all training sessions (including resistance training and cross-training) and matches. HWI was not considered a part of training sessions. Training load was calculated for all participants using the session RPE x time method using the CR-10 RPE scale [24].

#### Testing methods

The 30–15 intermittent performance test (30-15_IFT_) was used to assess the participants high intensity intermittent running performance (V_IFT_). The 30-15_IFT_ was conducted over two testing days for both pre- and post-intervention (4 sessions in total). All 30-15_IFT_ were conducted in an indoor basketball stadium within a 5-day period either side of the 6-week HWI intervention. For the pre-intervention testing, 13 of the 15 participants completed the 30-15_IFT_ in 27 °C and 27% RH environmental conditions, whilst the remaining two participants completed testing in 19 °C and 43% RH. For the post-intervention 30-15_IFT_, 14 of the 15 participants performed testing in 15 °C and 70% RH and one participant in 17 °C and 57% RH. A standardised warm-up protocol was completed prior to each 30-15_IFT_. All 30-15_IFT_ sessions were completed at a similar time (5:00 p.m. to 6:30 p.m.), with players encouraged to withhold from intense activity in the preceding 48 hours. All participants were familiar with intermittent running tests to exhaustion and completed a submaximal (ceased at 15 km·h^-1^) familiarisation session of the 30-15_IFT_ in the week prior to baseline testing.

Immediately prior to each testing session, all participants completed the same questionnaire relating to their beliefs about the intervention and how able they felt to perform testing. Specifically, these questions were “Do you believe the spa intervention will improve running performance?” and “What percentage of your full potential do you think you can run today?” An additional question “Do you believe your in-game running ability has improved since the start of the study?” was included during the Mid and Post time points. Answers to question one and three were scaled on a 11-point Likert Scale, ranging from 0 (Extremely Unlikely) to 10 (Extremely Likely), similar to methods used in previous HA studies [25].

A finger prick blood sample (100μL) was collected in week 1 (Pre), 4 (Mid) and after the final HWI session (Post; within 2 days of the final HWI session). Participants sat for approximately 10 min before and during collection. Haemoglobin (Hb) concentrations were determined in triplicate using a HemoCue^®^ Hb 20 (Hemocue AB, Ängelholm, Sweden). Haematocrit (Hct) was determined in duplicate via the microcapillary method; these samples were centrifuged at 12,000 rpm for 5 min. All samples were analysed within 10 min of collection. Percent change in plasma volume was calculated using hematocrit and hemoglobin values [26].

Body mass was measured to the closest 20g (PW-200-FC, A&D co., Tokyo, Japan) before and after each HWI session. Participants towel-dried themselves and donned dry underwear prior to measurement.

Tympanic temperature (Thermoscan, Braun GmbH, Kronberg, Germany), HR (Fingertip Pulse Oximeter, ChoiceMMED, Beijing, China) and thermal sensation (using a 7-point scale from -3 “unbearably cold” to 3 “unbearably hot”) [23] were recorded immediately pre and then at 5-min intervals during HWI sessions. The tympanic temperature recording device was stored at room temperature and was only exposed to the spa conditions for brief periods for recording. Test-retest reliability data is displayed for measures without a control group. Intraclass correlation coefficient (ICC) and coefficient of variation (CV) collected from our laboratories are as follows: tympanic temperature (ICC = 0.76, CV = 0.6%), HR (ICC = 0.92, CV = 4.3%) and thermal sensation (ICC = 0.71, CV = 10.6%).

### Statistical analyses

Data are presented as mean (standard deviation) unless stated otherwise. All data were assessed for normaility using Shapiro-wilk normaility tests, with non normally-distributed data log-transformed before analysis. P-values are reported, however, no level of significance was set, as per recommendations from recent American Statisitician journal editorials and American Association of Statistics position statement [27, 28]. Therefore, whilst p-values are reported in this study, effect size (Cohen’s d) is the predominant statistic variable used for assessing change. Cohen’s d (d) were calculated using the standard deviation of the mean difference for within-group changes, whilst between-group changes were calculated using the square root of average variance for between-group changes. Both between-group and within-group comparisons were calculated with 90% confidence intervals (90% CI). The following threshold values for effect size were employed: <0.2 as trivial, ≥0.2 as small, ≥0.5 as moderate, ≥0.8 as large [29]. Effect size was considered clear when 90% CI did not overlap both positive and negative values. Mixed-effects analyses were performed on all longitudinal data. Post-hoc testing was performed for changes between time points and independent sample t-tests were used for between-group changes. Statistical analyses were performed using Graphpad Prism 8 (version 8.3.1).

## Results

### Training load

A moderately lower total training load was seen in HEAT when compared to CON across the 6-week intervention (HEAT: 10,731 (1,408) AU vs CON: 11,488 (824) AU, d = -0.64 ± 0.88, p = 0.22), with an average weekly load of 1,795 (234) AU vs 1,915 (137) AU, respectively (d = -0.61 ± 0.87, p = 0.25). The difference between groups included large negative and small positive effects for both total (d = -1.51 to 0.24) and weekly load (d = -1.47 to 0.27).

### Hot water immersion

HWI exposure time across the 6 weeks was 322 (69) min (13 (2) sessions), with 222 (48) min exposure (8 (1) sessions) in the final three weeks of the intervention. HWI exposure resulted in large decreases in HR, tympanic temperature and thermal sensation during HWI between Initial to Final and Mid to Final timepoints (Table 1). When comparing Initial to Mid changes between-group, only thermal sensation resulted in a clear difference, with trivial changes seen in HR and tympanic temperature (Table 1).

**Table 1:** Physiological and perceptual data from Initial, Mid and Final hot water immersion sessions in HEAT.

	Initial (I) Mean (SD)	Mid (M) Mean (SD)	Final (F) Mean (SD)	% Change I vs M (Mean ± 90% CI)	% Change M vs F (Mean ± 90% CI)	% Change I vs F (Mean ± 90% CI)
**HR (bpm)**	110 (9)	108 (10)	101 (8)	-1.8 ± 7.4%	-6.6 ± 2.2%	-8.4 ± 6.4%
				d = -0.16 ± 0.59	d = -2.03 ± 1.05	d = -0.89 ± 0.70
				p = 0.66	p = 0.0007	p = 0.04
**Tympanic Temperature (°C)**	38.8 (0.4)	38.7 (0.3)	38.2 (0.4)	-0.2 ± 1.0%	-1.3 ± 0.7%	-1.4 ± 0.8%
				d = -0.11 ± 0.58	d = -1.26 ± 0.80	d = -1.18 ± 0.77
				p = 0.77	p = 0.009	p = 0.01
**TSS**	3.5 (0.5)	2.9 (0.6)	1.6 (0.5)	-18.9 ± 16.4%	-45.1 ± 16.6%	-55.5 ± 10.6%
				d = -0.70 ± 0.66	d = -1.35 ± 0.82	d = -2.30 ± 1.15
				p = 0.10	p = 0.005	p <0.0001

Note: HR: heart rate. bpm: beats per minute. TSS: Thermal sensation scale.

### Blood parameters (PV, Hct & Hb)

**Fig 2:**
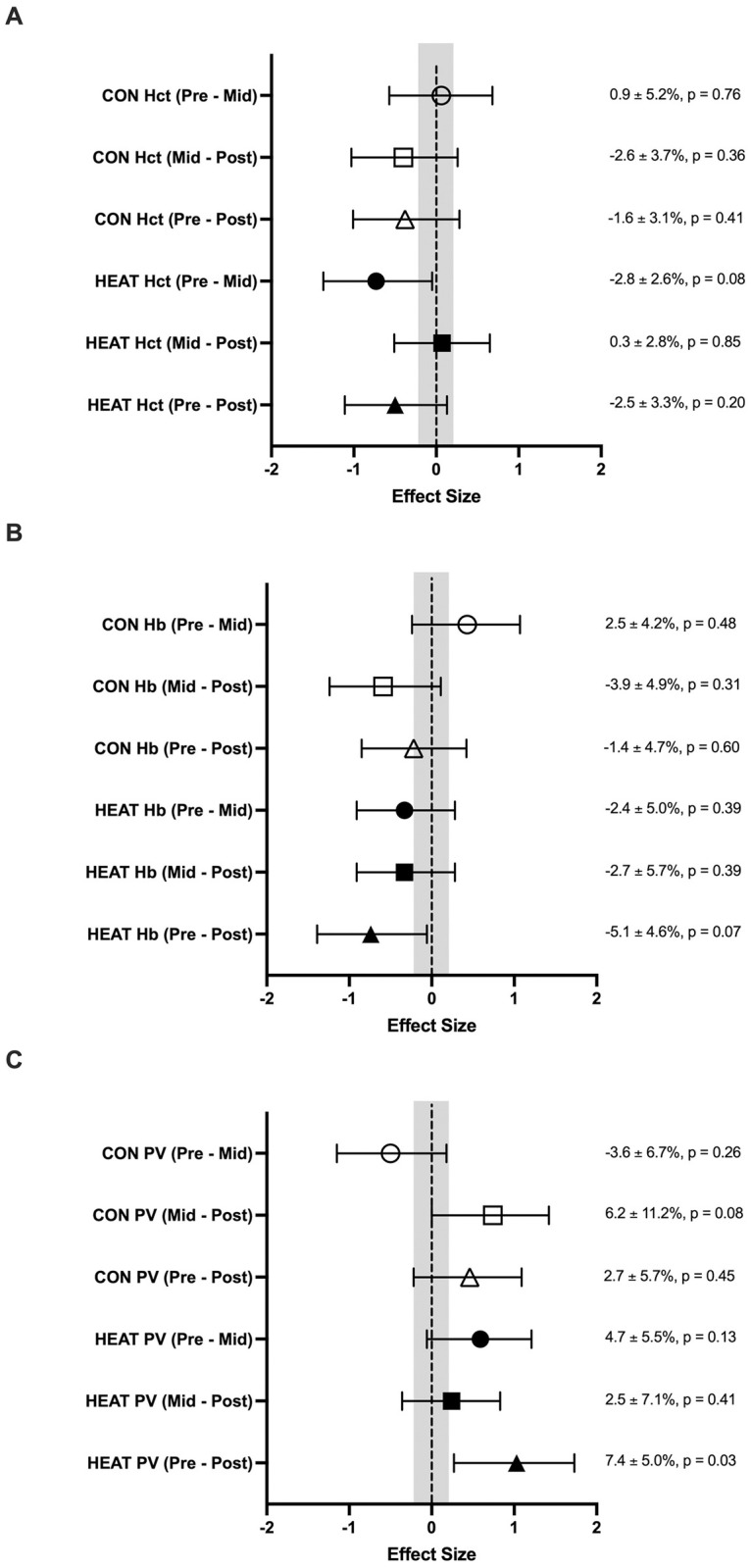
Within-group changes (Mean ± 90% CI) across the intervention (Pre, Mid, Post) for both HEAT and CON in Hb (A), Hct (B) and PV (C). Shaded area represents trivial change (d <0.2). The corresponding within-group % change and p-values are also presented.

Changes in blood parameters can be seen in Table 2 and Fig 2A–2C. Trivial to large changes were seen in blood parameters when compared within-group throughout the intervention (Fig 2A–2C). HEAT displayed a clear moderate decrease Pre to Mid in Hct (d = -0.73 ± 0.66). When comparing Pre to Post, a clear moderate decrease in Hb (d = -0.74 ± 0.66) and a clear large increase in PV (d = 1.03 ± 0.73) was seen in HEAT (Fig 2A–2C). The only clear change in blood parameters seen in CON was a moderate increase in PV from Mid to Post (0.74 ± 0.71) (Fig 2A–2C). All other within-group changes contained CI’s that crossed both positive and negative effects. Trivial to large changes were seen when comparing change between groups, with PV from Pre to Mid (1.09 ± 0.92) the only variable to possess clear differences (Table 2).

**Table 2:** Blood Parameter (Hb, Hct and PV) data from Pre, Mid and Post-intervention in HEAT and CON.

		Differences (%) in change observed between HEAT vs CON					
		Pre	Mid	Post	Pre vs Mid (Mean ± 90% CI)	Mid vs Post (Mean ± 90% CI)	Pre vs Post (Mean ± 90% CI)
**Hb**	HEAT	14.4 (1.1)	14.0 (1.2)	13.6 (1.2)	-5.0 ± 6.2%	1.3 ± 6.9%	-3.7 ± 6.2%
**(g/L)**	CON	14.3 (0.6)	14.7 (0.7)	14.1 (1.0)	d = -0.73 ± 0.88	d = 0.17 ± 0.85	d = -0.55 ± 0.87
					p = 0.17	p = 0.75	p = 0.30
**Hct**	HEAT	43 (2)	41 (2)	42 (3)	-3.7 ± 5.4%	2.8 ± 4.3%	-0.9 ± 4.2%
**(%)**	CON	42 (2)	42 (2)	41 (1)	d = -0.68 ± 0.88	d = 0.62 ± 0.87	d = -0.20 ± 0.85
					p = 0.24	p = 0.26	p = 0.71
**ΔPV**	HEAT		4.7 (5.5)	7.4 (5.0)	8.9 ± 7.9	-4.1 ± 8.6	4.4 ± 6.3
**(% from Pre)**	CON		-3.6 (6.7)	2.7 (5.7)	d = 1.09 ± 0.92	d = -0.39 ± 0.86	d = 0.63 ± 0.87
					p = 0.06	p = 0.42	p = 0.23

Hemoglobin. Hct: Hematocrit. PV: Plasma volume.

### Belief in HWI (V_IFT_)

Both HEAT and CON believed the HWI intervention would “likely” to “extremely likely” improve V_IFT_ when surveyed pre-intervention, with trivial differences seen between the groups both pre (HEAT: 6.8 (1.0) AU vs CON: 6.9 (1.5) AU, d = -0.09 ± 0.85, p = 0.88) and post-intervention (HEAT: 7.4 (1.4) AU vs CON: 7.3 (1.7) AU, d = 0.06 ± 0.85, p = 0.90). When comparing changes within-group from pre- to post-intervention, moderate and small increases were seen in HEAT (9.3 ± 10.5%, d = 0.59 ± 0.63, p = 0.29) and CON (6.3 ± 22.2%, d = 0.21 ± 0.63, p = 0.49), respectively. However, both within-group (HEAT and CON) changes contained CI’s that included both positive and negative effects. When compared between-group, trivial differences were seen (4.3 ± 28.2%, d = 0.12 ± 0.85, p = 0.83) from pre- to post-intervention.

### Perceived readiness

Between-group differences (HEAT vs CON) in self-rated ability to perform their best during performance testing were small or trivial at pre- (78 (6)% vs 76 (9)%, d = 0.23 ± 0.85, p = 0.98) and post-intervention (82 (9)% vs 83 (10)%, d = -0.10 ± 0.85, p = 0.97), respectively, with a small decrease in readiness seen between groups from pre- to post-intervention (-3.6 ± 12.2%, d = -0.27 ± 0.85, p = 0.61). All within and between-group changes contained CI’s that included both positive and negative effects.

### V_IFT_

**Fig 3:**
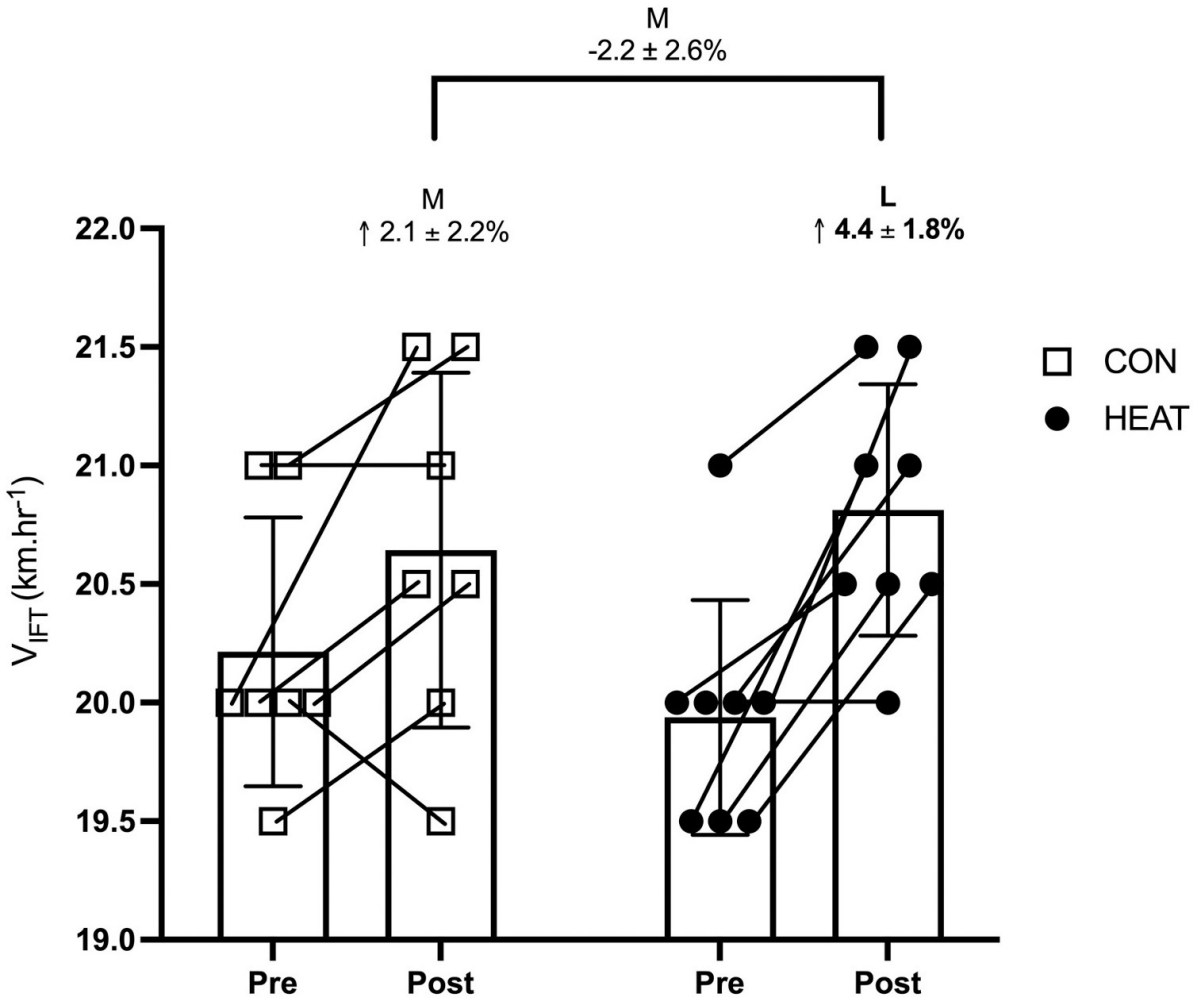
Mean ± SD 30–15 intermittent test (V_IFT_) final velocity pre- and post-intervention in HEAT and CON. Within-group changes are indicated above the respective post-value and above those is the between-group comparison of change. All comparisons are expressed as mean ± 90% confidence intervals. Letters depict effect size (d); L (large), M (moderate), with bold utlised to express clear differences.

When comparing V_IFT_ at Pre, HEAT was moderately lower than CON (d = -0.52 ± 0.87, p = 0.59), with differences between the groups including large negative and small positive effects (d = -1.39 to 0.35). When changes from pre to post-intervention were compared within-group, HEAT showed a clear large increase in VIFT (19.9 (0.5) km·h^-1^ vs 20.8 (0.5) km·h^-1^, d = 1.67 ± 0.93, p = 0.0007) whilst CON displayed a moderate increase (20.2 (0.6) km·h^-1^ vs 20.6 (0.8) km·h^-1^, 2.1 ± 2.2%, d = 0.65 ± 0.90, p = 0.07). However, changes in CON included effects that crossed both positive and negative values. Between-group comparisons revealed a moderately larger increase in V_IFT_ in HEAT (2.2 ± 2.6%, d = 0.81 ± 0.89, p = 0.15) when compared to CON (Fig 3), with differences between groups including trivial negative and large positive effects (d = -0.09 to 1.69).

### In-game running performance belief

**Fig 4:**
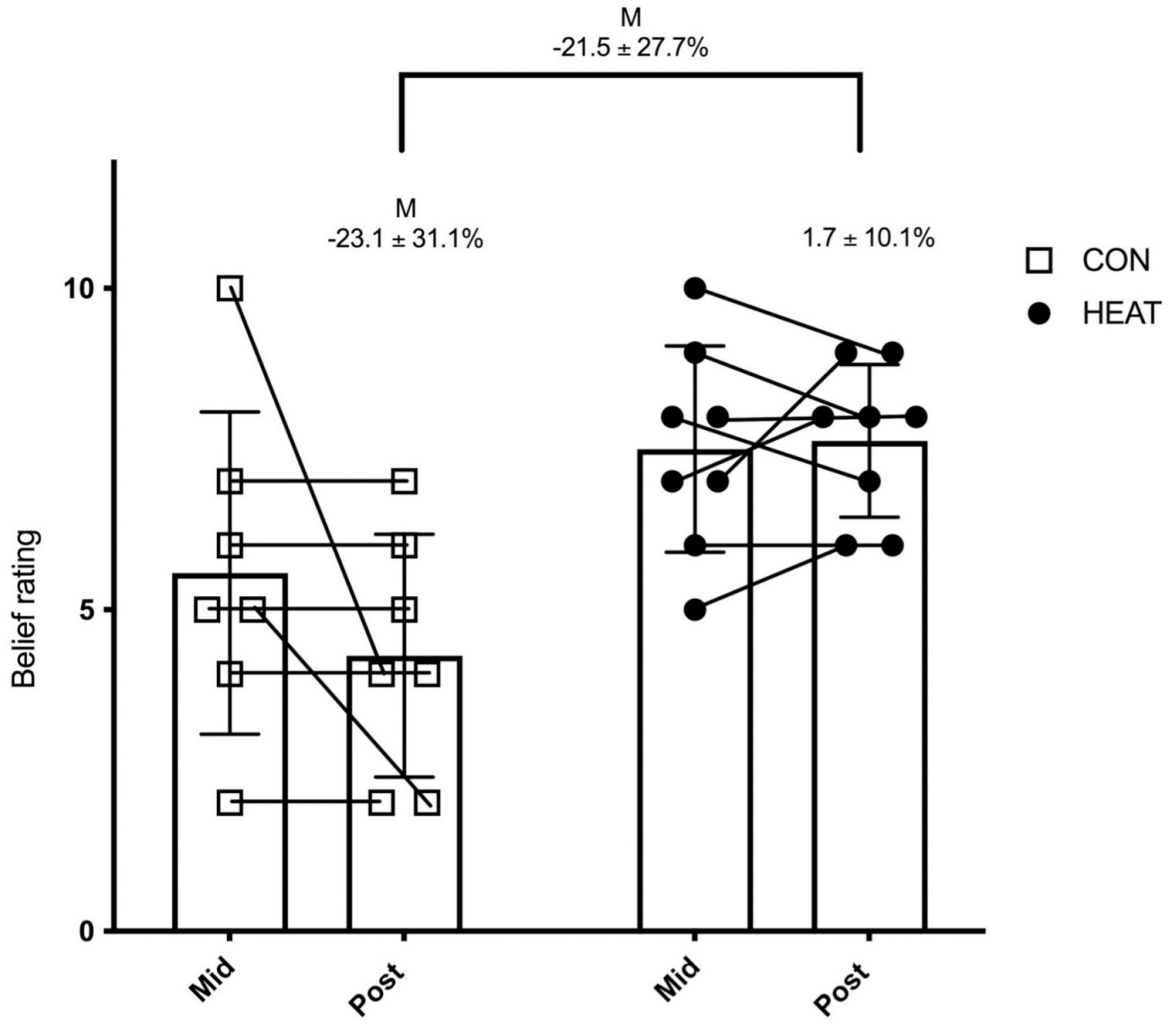
Belief (mean ± SD) that in-game running performance increased in HEAT and CON at mid and post-intervention time points. Within-group changes are indicated above the respective post-value and above those is the between-group comparison of change. All comparisons are expressed as mean ± 90% confidence intervals. Letters depict effect size (d); M (moderate).

HEAT believed that their in-game running performance “likely” to “extremely likely” increased at both mid- (7.5 (1.6) AU) and post-intervention (7.6 (1.2) AU), whilst CON’s belief remained “unchanged” (5.6 (2.4) AU) at mid-intervention and “unchanged” or “unlikely” increased (4.3 (1.9) AU) post-intervention (Fig 4). Large positive differences were seen in HEAT when compared to CON when examining the participants belief that their in-game running performance had improved at both mid- (29.2 ± 30.7%, d = 0.92 ± 0.89, p = 0.05) and post-intervention (55.0 ± 25.0%, d = 2.11 ± 1.06, p = 0.002). When changes in belief from mid- to post-intervention were compared within-group, both HEAT and CON showed trivial (d = 0.11 ± 0.58, p = 0.85) and moderate (d = -0.58 ± 0.89, p = 0.08) changes, respectively (Fig 4). When mid to post-intervention changes were compared between-group (HEAT vs CON), a moderately greater increase (d = 0.77 ± 0.88, p = 0.19) was seen in belief that in-game running performance had improved (Fig 4). All within and between-group differences included effects that crossed negative and positive values.

## Discussion

The aim of this current study was to investigate a) whether a longer-term (13 ± 2 session over a 6-week period) passive HWI protocol could elict physiological adaptations consistent with HA, and b) if such a protocol could improve intermittent running performance and athletes’ belief in within-match running ability during the in-season (six matches were performed during the intervention period). This study is the first, to the authors’ knowledge, to demonstrate that passive HWI may be of benefit in-season for team sport athletes. The findings of this study show the addition of HWI 2–3 x/week to a semi-professional ARF training program can induce HA and increase belief in match running performance. Furthermore, HWI may have the potential to improve intermittent running performance when compared to team-training alone, amongst semi-professional ARF athletes. This potential was evidenced via large improvements in V_IFT_ for HEAT (d = 1.67), with effects ranging from trivial decreases to large increases in V_IFT_ (d = 0.81, CI: -0.09 to 1.69) when compared between HEAT and CON.

Our study illustrates that some signs of HA, as evidenced by a moderate decrease in thermal sensation (d = -0.70) in HEAT during HWI, as well as a large increase in PV when compared to CON, may occur after as little as five HWI exposures across a 3-week period (total exposure time = 110 min). To date, no study has investigated such a dispersed HWI acclimation protocol, with previous HWI protocols utilising either continuous or alternate-day protocols of 6 or more sessions. For example, Zurawlew and colleagues [11] have previously shown adaptations such as reduced physiological and perceptual strain during both HWI and submaximal exercise after six consecuitve days of HWI, whilst other alternate-day HWI studies ranging from 14 days (7 sessions) [9] to 21 days (10 sessions) [10] have shown signs of HA such as decreased resting core temperature and increased sweat response [9, 10]. With this in mind, the present study is the first to the authors knowledge to show that a passive HWI stimulus of approximately twice per week (~37 min exposure/week) over a 3-week period can illicit signs of HA.

Although signs of HA may occur following five sessions of HWI across a 3-week period, our findings suggest a longer (6-week) protocol achieves greater physiological adaptations consistent with further HA. Signs of continued adaptation were demonstrated via greater changes in HEAT for three (HR, tympanic temperature and thermal sensation) out of the four markers of HA in our study, with PV the only HA marker to not show a clear increase from mid- to post-intervention. It should be noted that amassed HWI time increased to 212 min in the final 3-week period (Mid to Post) from 110 min over the initial 3-week period (Pre to Mid). The lower HWI exposure time in the initial 3 weeks of the intervention was a deliberate approach to ensure a gradual increase in heat stress applied to the athletes, particularly as the study was completed in-season—a time when practitioners, coaches and athletes are most concerned about additional stressors. To determine an optimal in-season HA protocol, future work is required to compare the response to different exposure durations, such as those utilised between the first and second half of the present study. Specifically, if differing HWI protocols illicit greater changes to key HA markers (e.g., PV, core temp, HR) and performance measures such as intermittent running performance.

Our findings show the use of a 6-week HWI protocol improved intermittent running performance (V_IFT_) in HEAT, with preliminary evidence suggesting the addition of HWI may be of benefit to V_IFT_ when added to a team-sport training program. This was evidenced by a largely greater increase in V_IFT_ in HEAT (d = 0.81) when compared to CON. Although a large increase in V_IFT_ when compared to CON is promising, it is important to note that a small sample size was used in this study, therefore, leading to large variability in the effects a practitioner may expect to see (d = -0.09 to 1.69). Despite the large variability seen in the effect of HWI on V_IFT_ in our study, the 90% CI ranges from trivial decreases to large increases, suggesting the potential benefits associated with the addition of HWI outweight the possible detrimental effects.

When interpreting individual changes in V_IFT_ seen in this study, it is worth considering the unavoidable differences in environmental testing conditions, pre- vs post-intervention, with a difference of up to 12 °C (27 °C vs 15 °C) between the testing conditions for the majority of participants. Previous literature suggests temperature differences of a similar nature may impact running performance [11, 30]. Therefore, the authors recommend utilising between-group comparisons for assessing the impact of the intervention, as both groups (HEAT and CON) experienced comparable changes in testing environments.

To date, there are currently only six studies [2–7] that have investigated the effectiveness of HA on intermittent running performance in team sport environments, with the findings seen in our study comparable to the previous literature. These studies [2–7] showed effects of 0.5–44% (d = 0.0–2.0) on intermittent running performance, however, the majority of previous literature that show improvements in running performance [2–5] have lacked a control group [2, 3, 5]. Of the three prior studies [4, 6, 7] to utilise a control group, only Sunderland et al. [4] showed the effect of HA on intermittent running performance to be greater than trivial (ES > 0.2), as seen in the present study. It is worth noting the three studies [4, 6, 7] utilised much shorter HA protocols (≤5 sessions) than the current study, with performance testing completed in temperate conditions (18–24°C) in the Philp et al. [6] and Petersen et al. [7] studies, but not Sunderland et al. [4] (31°C). Although notable differences in intervention modality (HWI vs running [4] vs cycling [6, 7]) and testing protocol (30-15_IFT_ [6] vs repeated sprint [7]) make it difficult to explain why differences were seen between previous literature [4, 6, 7], it may be suggested that while short-term HA can improve intermittent running performance in the heat, longer acclimation periods may be required to elicit improvements in temperate conditions. Therefore, given that it can be implemented without additional training load, our findings present passive HWI as a stimulus warranting further investigation of its impact on intermittent running performance in semi-professional athletes during the in-season competition period.

While there are increased physiological stresses associated with HA protocols, the passive modality of our intervention avoids increasing training load during the season and disruption the football program; concerns often harboured by coaches and practitioners. A potential limitation of previous team sport HA protocols, and a possible explanation for why HA is not frequently used by team sports, is that team training is either performed in the heat [2, 3], or active HA is prescribed as an addition to the training program [6, 31], resulting in increased training loads. Furthermore, some of the more effective team sport HA studies required international travel [2, 3]. Active HA protocols pose disruption to the football program by way of either logistics such as travel [2, 3] or increased training load and intensity via additional active HA sessions or team training in the heat [6, 31]. A benefit of the present study is that the HA protocol was performed after training, in the normal club training environment without increasing logistical demands, training volume or intensity. Consequently, the passive HA protocol used in the present study minimised disturbances to the football program to maximise skill and tactical training exposure, whilst improving athlete self-reported in-game running ability belief, and potentially improving intermittent running performance, during the in-season competitive period.

All athletes held positive perceptions that HWI could lead to improved performance in the present study. These beliefs may be a contributing factor explaining the potential improvements in intermittent running performance in our study, as both HEAT and CON believed the HWI would improve intermittent running performance. The ability for belief to affect exercise performance is not new, with several examples of this occurring with other interventions such as caffeine [16], bicarbonate [17], cold water immersion [18], and altitude training [15]. For example, McClung and Collins [17] found a significant positive effect on 1,000m running performance in well-trained middle distance athletes when told they were consuming sodium bicarbonate supplementation, irrespective of whether they received sodium bicarbonate or a placebo. Whereas, no benefit to running performance was observed when athletes were told they weren’t consuming sodium bicarbonate, despite being administered the supplement [17]. Thus, suggesting a strong effect of belief on intervention-derived performance outcomes. Despite previous examples seen with other ergogenic aids, our study is the first to the authors’ knowledge to investigate the belief effect of HWI on intermittent running performance in semi-professional ARF athletes.

Hot water immersion was shown to improve athletes’ perception of in-game running performance, with HEAT believing their in-game running ability largely improved post-intervention (d = 2.15) when compared to CON. This finding is arguably equally or more important than both the potential increases in intermittent running performance and belief that HWI would improve intermittent running performance seen in our study. Belief in an intervention is a direct measure of how the athlete feels the intervention impacts on their competition performance, not a moderator or associated measure such as increased intermittent running performance [1]. Secondly, there is evidence suggesting that when athletes feel more prepared, thus, possess high levels of self-confidence, the potential for positive performance outcomes increases [32]. With this in mind, our study offers a novel insight in to the psychological benefits of HWI interventions that have the potential to influence perceptions of running ability during competition in semi-professional ARF athletes, after as little as 5 exposures, and even more so after 13 exposures.

Relative to previous passive HA interventions, the passive HA protocol used in the present investigation was often performed after either resistance training or team meeting commitments, not directly after aerobic endurance training. Scoon et al. [33] and Zurawlew et al. [11] implemented their heat exposure as soon as possible after aerobic exercise in an attempt to prolong both the elevated core temperature and cardiovascular stimulus of the preceding session. This is often not possible in the team sport environment, due to competing time demands between physical and tactical priorities. Our study suggests that performing passive heat exposure directly after aerobic exercise may not be required, at least in the team sport athlete. In our study, participants were still experiencing tympanic temperatures >38 °C for at least 15 min during the final HWI session, ultimately leading to adaptations consistent with HA. These findings further highlight the practicality of HWI as part of a high performance program due to the flexibility of implementatition relative to the team schedule.

The authors acknowledge the limitations of tympanic temperature in determining core temperature changes. However, tympanic temperature measurements provide an indication of temperature change within the athletes in a less invasive method than either rectal or ingestible core temperature pills. With the latter deemed unsuitable for the cohort used in this study given the collision nature of the sport, and therefore, the high chance of imaging tests such as MRI needing to be performed. The constraints of a contact sport competitive season limited the ability to implement the HWI as initially planned (15 sessions across the 6-week intervention period); some players missed sessions. This was largely due to team medical practitioners removing participants from HWI sessions due to injuries such as contusions. However, injuries such as contusions are a regular occurrence in collision-based sports, therefore, adding ‘real-world’ context to our findings. Lastly, the authors acknowledge the small sample size used in this study as a limitation.

Despite the promising novel results of the present study, future post-exercise HWI studies involving greater participants to allow clearer interpretations and elite level teams would be of significant value. Elite level athletes are found to already posses many adaptations consistent with HA [22], thus, have traditionally shown less room for HA adaptation than trained individuals [34]. Therefore, whether a passive HA modality of 30 min or less can elicit adaptions and performance increases associated with HA in elite team sport athletes remains to be seen and caution should be taken when applying the present results to higher-level team-sport athletes.

In conclusion, passive HA from post-exercise HWI (13 sessions, 322 min across a 6-week period) can elicit HA and increase athletes perception of in-game running ability in-season in semi-professional ARF athletes. Further investigation is required to determine whether HWI improves intermittent running performance to a greater extent than normal training alone.
